# Human serum RNase-L level is inversely associated with metabolic syndrome and age

**DOI:** 10.1186/s12933-017-0522-x

**Published:** 2017-04-11

**Authors:** Yi-Ting Wang, Ping-Huei Tseng, Chi-Ling Chen, Der-Sheng Han, Yu-Chiao Chi, Fen-Yu Tseng, Wei-Shiung Yang

**Affiliations:** 1 Graduate Institute of Clinical Medicine, College of Medicine, National Taiwan University, No. 7, Chung-San South Road, Taipei, 10002 Taiwan; 2 Division of Gastroenterology, Department of Internal Medicine, National Taiwan University Hospital, No. 7, Chung-San South Road, Taipei, 10002 Taiwan; 3 Graduate Institute of Epidemiology and Preventive Medicine, College of Public Health, National Taiwan University, No. 17, Xu-Zhou Road, Taipei, 10055 Taiwan; 4 Department of Physical Medicine and Rehabilitation, National Taiwan University Hospital Beihu Branch, No.87, Neijiang St., Taipei, 10800 Taiwan; 5 Community and Geriatric Medicine Research Center, National Taiwan University Hospital Beihu Branch, No.87, Neijiang St., Taipei, 10800 Taiwan; 6 Division of Endocrinology & Metabolism, Department of Internal Medicine, National Taiwan University Hospital, No. 7, Chung-San South Road, Taipei, 10002 Taiwan; 7 Center for Obesity, Lifestyle and Metabolic Surgery, National Taiwan University Hospital, No. 7, Chung-San South Road, Taipei, 10002 Taiwan; 8 Graduate Institute of Medical Genomics & Proteomics, College of Medicine, National Taiwan University, No.1, Sec. 1, Jen-Ai Road, Taipei, 10051 Taiwan; 9 No. 1, Chang-Teh St., Taipei, 10048 Taiwan

**Keywords:** RNase-L, Metabolic syndrome, Central obesity, Elevated blood pressure, Impaired fasting glucose, Low high-density lipoprotein cholesterol

## Abstract

**Background:**

Ribonuclease-L (RNase-L) was known to be a ubiquitous enzyme involved in several cellular functions, especially innate immunity. It was recently shown to participate in adipogenesis in rodents. Here, we developed a method to measure serum levels of RNase-L and analyzed the relationship between RNase-L and metabolic syndrome (MetS).

**Methods:**

A total of 396 subjects were recruited from a health check-up program. An in-house RNase-L immunoassay was developed. The serum RNase-L levels of these subjects were measured, and the association of MetS-related factors with RNase-L levels was assessed.

**Results:**

The mean serum level of RNase-L of the subjects with MetS were lower than those without (16.5 ± 6.4 vs. 18.4 ± 8.0 μg/ml, *P* = 0.018). The subjects with central obesity, elevated blood pressure, or impaired fasting glucose also had lower serum RNase-L levels in comparison to those without. In multivariate linear regression analysis, diastolic blood pressure (β = −0.129, *P* = 0.024) and high-density lipoprotein cholesterol (HDL-C) (β = 0.127, *P* = 0.036) were related to serum RNase-L. For every 5 μg/ml increase in serum RNase-L levels, it is associated with a reduced risk of MetS (OR 0.83, 95% CI 0.71–0.98, *P* = 0.028), central obesity (OR 0.82, 95% CI 0.71–0.94, *P* = 0.005), or low HDL-C (OR 0.86, 95% CI 0.74–1.00, *P* = 0.042). Moreover, age is inversely related to serum RNase-L levels in various analyses.

**Conclusions:**

The serum RNase-L levels were inversely associated with MetS, unfavorable metabolic profiles, and age.

## Background

Over the past decades, the prevalence of metabolic syndrome (MetS) has been increasing worldwide [1]. The cardiometabolic disorders associated with the MetS, such as obesity, diabetes mellitus (DM), dyslipidemia, hypertension, and cardiovascular diseases impose a huge burden both socially and economically on many developed and developing countries [2]. Our understanding about the pathogenic mechanisms underlying the MetS has also been advanced in the past decades [3–5]. Nowadays, it is clear that the full spectrum of the manifestations in MetS is not simply metabolic. In addition to the traditional metabolic factors, inflammation also plays important roles in the pathogenesis and the complications of these metabolic disorders [6, 7]. A chronic low-grade inflammation that may arise from environmental and genetic factors, is likely involved in the development of insulin resistance and atherosclerosis in the MetS [8, 9]. In view of this, several inflammatory indicators, such as C-reactive protein, tumor necrosis factor-α, and interleukin-1β have been shown to be potential biomarkers for the MetS [7, 10, 11].

In mammals, ribonuclease L (RNase-L) was originally recognized as a key factor in response to viral infection in innate immunity. It belongs to the interferon (IFN)-oligoadenylate synthetase (OAS)-RNase L. OASs are first activated by viral double-stranded RNA and therefore produce 2-5A (ppp5′A[2′p5′A]n) from ATPs. RNase-L is then activated by 2-5A. The activated RNase-L partially degrades the invading viral RNA and cellular RNA as well. Retinoic acid-inducible gene 1 (RIG-1), in turn, is activated by these cleavage RNAs, and the downstream signals trigger the production of inflammatory cytokines, such as type I interferon (IFN) [12]. However, the role description of RNase-L was recently expanded. It was reported to regulate a wide variety of biological functions, by degrading specific cellular mRNA through its ribonuclease activity in different tissues [13, 14]. The mRNA substrate list of RNase-L includes myogenic differentiation-1 (MyoD) and myogenin for myogenesis, and Hu Antigen R (HuR) and tristetraprolin (TTP) for cell proliferation [15–19]. We and others also showed that RNase-L participates in adipogenesis via regulating pre-adipocyte factor-1 (Pref-1) and CCAAT/enhancer-binding (CEBP) homologous protein 10 (CHOP10) [20, 21]. The animal with RNase-L knockout however had increased adipose tissue with ectopic fat deposition in liver and kidney [21]. In addition, over-expression of RNase-L in mouse and human myotubes were demonstrated to improve insulin signaling in a palmitate-induced insulin resistance model [22]. Interestingly, the myotubes from obese insulin resistant humans appeared to have defective RNase-L activation [22].

Taken together, RNase-L may play a role in the pathogenesis of human metabolic disorders. We developed an in-house enzyme-linked immunosorbent assay (ELISA) to measure human serum RNase-L levels and relate its blood concentration to various metabolic factors in human subjects.

## Methods

### Study subjects

This study was approved by the Research Ethical Committee of National Taiwan University Hospital (NTUH, No. 201204030RIB), following the established guideline. A total 524 of subjects who participated in a self-paid health check-up program at the Health Management Center of NTUH with individual informed consent were recruited. The information of their anthropometric measurements, biochemical data, medical history and medication usage were documented. Subjects with acute illness, malignancy, chronic inflammation, infection, and more extreme metabolic conditions, including rheumatoid arthritis, systemic lupus erythematosus, chronic hepatitis B, chronic hepatitis C infection, current users of anti-thyroid, glucocorticoids, unclear cold medicine, and non-steroid anti-inflammatory drugs were excluded. Finally, 396 ostensibly healthy subjects were recruited for this study.

The anthropometric measurements were as previously described [23]. In addition, the mass and percentage of body fat were determined by the body composition analyzer DX-300 (Jawon Medical, Gyeongsan, Korea). The fasting blood samples were collected after at least 8-h overnight fasting. The blood chemistry, including fasting blood glucose, triglycerides, cholesterol, and so on, was assayed as previously described [23].

The modified criteria of metabolic syndrome (MetS) from the International Diabetes Federation (IDF) and the American Heart Association/National Heart, Lung, and Blood Institute (AHA/NHLBI) for Asian were applied: (1) central obesity: waist circumference ≥90/80 cm in male/female; (2) elevated blood pressure: blood pressure (BP) ≥ 130/85 mmHg [systolic(SBP)/diastolic(DBP)], diagnosed hypertension, or with hypertension treatment; (3) low high-density lipoprotein cholesterol (HDL-C): HDL-C < 40/50 mg/dl in male/female; (4) Impaired fasting glucose (IFG): fasting glucose ≥ 100 mg/dl, diagnosed type 2 diabetes, or with anti-diabetic treatment; (5) hypertriglyceridemia: triglycerides (TG) ≥ 150 mg/dl [24–27]. MetS was diagnosed with at least three of the above five criteria.

### Development of the immunoassay for human serum RNase-L

We developed an indirect enzyme-linked immunoassay (ELISA) to measure human serum RNase-L concentration [28]. Initially, the stock of human full-length RNase-L recombinant protein (Origene, Rockville, USA, TP314849) was diluted to the concentration of 200 ng/ml with ice-cold PBS, and then 100 μl of diluted RNase-L solution was added into each well of a Nunc MaxiSorp^®^ flat-bottom 96-well plate (ThermoFisher, Waltham, USA, 44-2404-21), covered and incubated at 4 °C overnight with gently rocking (50 rpm) on a horizontal shaker. The solution was removed and the plate was washed three times with 300 μl of 1× Tris-buffered saline with 0.05% (v/v) Tween-20 (TBST). The plate was rigorously blotted on paper towel until the fluid residue was entirely removed. One hundred μl of blocking buffer [TBST containing 1% (w/v) BSA] was added to each well. The plate was covered and incubated at 4 °C overnight with gentle shake as above. On the day of assay, the blocking buffer was removed and the plate was blotted dry.

Fifty μl of full-length recombinant RNase-L protein solution with a final concentration (f.c.) respectively of 16, 8, 4, 2, 1, 0.5 and 0.25 μg/ml after serial dilution with blocking buffer and one blank solution with blocking buffer only were used to generate a standard curve. Fifty microliter of serum samples (diluted with blocking buffer by two or fourfold) were added to each well for assay. The plate was incubated at 37 °C with horizontally shaking (150 rpm) for 1 h. A rabbit polyclonal antibody against human RNase-L (Proteintech, Chicago, USA, 22577-1-AP) was diluted to the working concentration (1/2000 dilution, f.c. = 146.7 ng/ml) with blocking buffer and 50 μl was added into each well, incubated for another hour. The mixture was discarded and the plate was washed thrice with TBST and blotted dry. A goat horseradish peroxidase (HRP)-conjugated anti-rabbit IgG polyclonal antibody (GeneTex, Irvine, USA, GTX213111-01, 1/5000 dilution, f.c. = 75 ng/ml, 100 μl) was next added into each well and incubated for 1 h in the same condition. The plate was then washed five times with TBST and blotted dry. Then 100 μl of 3, 3′, 5, 5′-tetramethylbenzidine (TMB) substrate for HRP was added and incubated without shaking for 10 min at room temperature. During this coloration step, plate was placed in a dark drawer to avoid light exposure. To terminate color development, 100 μl of 1 M sulfuric acid (Merck, Darmstadt, Germany) was added. The optical density (OD) of each well was determined by the ELISA reader (VersaMax™ ELISA Microplate Reader, Molecular Devices, Sunnyvale, California) at 450 nm wavelength. The corresponding RNase-L levels of the standards with their OD values were plotted to generate a standard curve using 4-parametric logistic regression. An R^2^ ≥ 0.99 was acquired. For validation of the ELISA, determination of inter- and intra-assay coefficients of variability (CV) and the analytical sensitivity were basically performed as previously described [28, 29]. The CV (%) of each replicates were calculated using the following formula, (SD of replicates)/(mean of replicates) × 100%. To analyze intra-assay CV (%), duplication of 16 samples with different concentrations were run in an assay. The intra-assay CV (%) calculated from the average CV (%) of each duplicates was 4.7%. To analyze inter-assay CV (%), 8 samples with different concentration were measured in 7 independent assays. The inter-assay CV (%) calculated from the average CV (%) of each septuplicates, was 7.0%. The analytical sensitivity of our serum RNase-L ELISA assay was calculated by that the mean of assay results for the 9 zero standard replicates subtracted twofold standard deviations (SD) of the mean. The limit of detection of our ELISA was 0.52 μg/ml.

### Statistical analysis

The statistically significant differences of selected anthropometric measurements, biochemical characteristics and RNase-L levels between those with and without metabolic syndrome were tested by *t* test for continuous variables and Chi square test for categorical variables. Linear regression model with the serum RNase-L levels as dependent variable was used to evaluate the direction and strength of the selected factors. For categorical variable, the dose–response trend was evaluated by treating the categorical variables as group linear in the linear regression model. Logistic regression models were performed with the adjustment of age and gender to examine the odds ratios (OR) with 95% confidence intervals (CI) to estimate the association of every 5 μg/ml increase in serum RNase-L levels in relation to MetS or individual component of MetS. All statistical analyses were conducted using IBM SPSS statistics 22.0 (IBM Corporation, Armonk, USA) and the two-tailed *P* value <0.05 is considered significant.

## Results

In a total of 396 subjects, the mean age was 53.3 years old and 60.4% were male. One hundred and four of them (26.3%) had metabolic syndrome (MetS) (Table 1). There were more males in MetS group (Table 1). As expected, the subjects with MetS had higher body weight, body mass index (BMI), waist circumference, mass and percentage of body fat, fasting blood glucose, hemoglobin A1c (HbA1c), BP and TG, but lower HDL-C in comparison to those without (Table 1). These MetS subjects were also with a higher percentage of the medical history of hypertension, diabetes mellitus, or hyperlipidemia (Table 1). Interestingly, we found that the mean RNase-L level of the MetS group was significantly lower than that of non-MetS (16.5 ± 6.4 vs. 18.4 ± 8.0 μg/ml, *P* = 0.013, Table 1).

**Table 1:** Demographic and biochemical characteristics of the subjects with or without metabolic syndrome (MetS)

Variables	non-MetS	N	MetS	N	*P* value	Total	N
	Mean ± SD		Mean ± SD			Mean ± SD	
Demographics							
Age (year)	52.9 ± 9.9	291	54.3 ± 8.2	105	0.206	53.3 ± 9.5	396
Gender (% male)	55.0%	291	75.2%	105	<*0.001*	60.4%	396
Body weight (kg)	62.3 ± 10.6	290	76.1 ± 13.3	104	<*0.001*	66.0 ± 12.9	394
BMI (kg/m^2^)	23.1 ± 2.8	290	27.2 ± 4.3	104	<*0.001*	24.1 ± 3.8	394
Waist (cm)	83.3 ± 8.0	291	94.5 ± 8.5	105	<*0.001*	86.2 ± 9.6	396
Percentage of body fat (%)	25.6 ± 5.2	290	29.4 ± 4.3	103	<*0.001*	26.6 ± 5.3	393
Mass of body fat (kg)	16.1 ± 4.7	290	22.3 ± 5.4	103	<*0.001*	17.7 ± 5.6	393
Systolic blood pressure (mmHg)	115.4 ± 12.9	291	129.3 ± 17.0	105	<*0.001*	119.1 ± 15.4	396
Diastolic blood pressure (mmHg)	69.1 ± 9.4	291	79.3 ± 11.9	105	<*0.001*	71.8 ± 11.0	396
Pulse pressure (mmHg)	46.4 ± 8.0	291	50.1 ± 8.8	105	<*0.001*	47.7 ± 10.2	396
Mean arterial pressure (mmHg)	84.5 ± 10.0	291	95.9 ± 13.2	105	<*0.001*	87.7 ± 12.0	396
Blood chemistry							
Fasting glc (mg/dl)	93.1 ± 13.2	291	110.8 ± 35.9	105	<*0.001*	97.8 ± 23.0	396
HbA1c (%)	5.6 ± 0.4	291	6.2 ± 1.2	105	<*0.001*	5.7 ± 0.8	396
Triglyceride (mg/dl)	100.2 ± 48.8	291	198.8 ± 132.2	105	<*0.001*	126.3 ± 90.8	396
Total cholesterol (mg/dl)	195.8 ± 34.7	291	195.6 ± 37.9	105	0.975	195.8 ± 35.5	396
HDL-C (mg/dl)	54.4 ± 12.4	291	40.9 ± 9.0	105	<*0.001*	50.8 ± 13.1	396
LDL-C (mg/dl)	121.9 ± 30.0	291	122.3 ± 31.2	105	0.867	122.0 ± 30.2	396
TP (g/dl)	7.2 ± 0.4	291	7.3 ± 0.4	105	0.357	7.3 ± 0.4	396
Medical history and drug usage							
Hypertension (%)	13.4%	291	46.2%	105	<*0.001*	22.0%	396
Diabetes (%)	4.1%	291	21.2%	105	<*0.001*	8.6%	396
Hyperlipidemia (%)	9.2%	291	29.8%	105	<*0.001*	14.6%	396
Drug for elevated BP (%)	12.0%	291	45.2%	105	<*0.001*	20.7%	396
Drug for elevated glc (%)	4.8%	291	18.3%	105	*0.001*	8.3%	396
Drug for elevated TG (%)	8.2%	291	21.2%	105	*0.003*	11.6%	396
RNase-L (μg/ml)	18.4 ± 8.0	291	16.5 ± 6.4	105	*0.013*	17.9 ± 7.6	396

*P* value represented the significance of independent t-tests between non-MetS and MetS

**Fig. 1:**
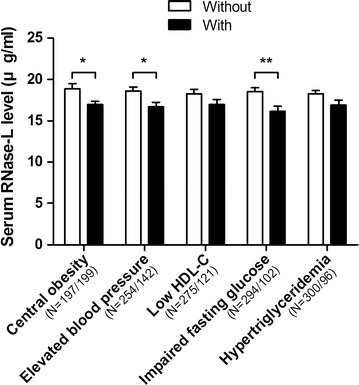
Comparison of serum RNase-L levels between the subjects with and without MetS components. The RNase-L levels of the subjects with and without the components were respectively shown with *black* and *white bars*. The data were shown as mean and SE. *P < 0.05 and **P < 0.01

**Fig. 2:**
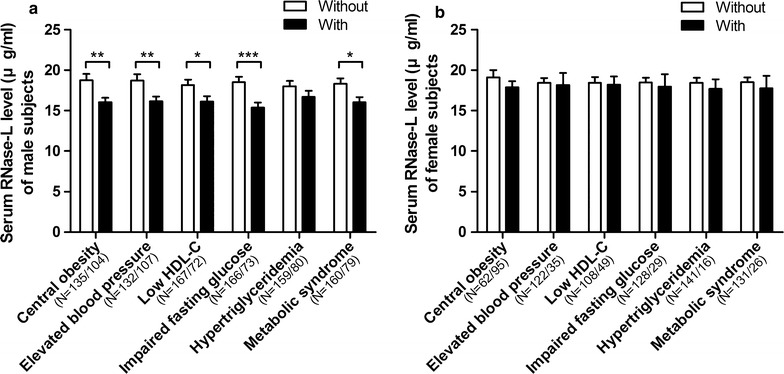
Gender-dependent comparisons of serum RNase-L levels between the subjects with and without MetS components. The differences of RNase-L levels between the (**a**) male or (**b**) female subjects with and without the components were respectively shown with *black* and *white bars*. The data were shown as mean and SE. *P < 0.05, **P < 0.01, ***P < 0.001

When further examining the serum RNase-L levels in the subjects categorized by each component of the MetS criteria, we found that the serum RNase-L concentration in general is lower in the subjects with worse cardiometabolic profiles than in those without (Fig. 1). The subjects with central obesity (16.9 ± 6.6 vs. 18.9 ± 8.4 μg/ml, *P* = 0.011), elevated BP (16.6 ± 6.9 vs. 18.6 ± 7.9 μg/ml, *P* = 0.015) and IFG (16.1 ± 6.5 vs. 18.5 ± 7.9 μg/ml, *P* = 0.003) respectively had lower serum RNase-L concentrations than those without (Fig. 1). Although the subjects with low HDL-C (17.0 ± 6.3 vs. 18.3 ± 8.1 μg/ml, *P* = 0.086) and hypertriglyceridemia (16.9 ± 6.3 vs. 18.2 ± 8.0 μg/ml, *P* = 0.134) also had lower serum RNase-L concentrations respectively than those without, the differences were not statistically significant (Fig. 1). However, if subjects were further separated by gender, these significant differences were only observed in male subjects, but not in females (Fig. 2). Moreover, there was a descending trend of RNase-L levels associated with increasing number of MetS components (β = −1.01, 95% CI = −1.55–0.47, *P* for trend <0.001).

In linear regression analyses with the adjustment of age and gender, we found that waist circumference, body fat percentage, DBP, mean arterial pressure (MAP), fasting glucose, HbA1c were independently related to the serum levels of RNase-L in a negative manner (Table 2). On the other hand, HDL-C was positively related to serum RNase-L level (Table 2, Model 7). In contrast, its relation with TG was of a borderline significance (Table 2, Model 10), whereas those with BMI and SBP were not significant (Table 2, Model 1 and 4).

**Table 2:** The relation between serum RNase-L and metabolic factors in multivariate linear regression analyses

Variables	Model 1 β (SE) *P* value	Model 2	Model 3	Model 4	Model 5	Model 6	Model 7	Model 8	Model 9	Model 10	Model 11
Age (year)	−0.123 (0.041)	−0.118 (0.040)	−0.114 (0.040)	−0.111 (0.041)	−0.114 (0.040)	−0.111 (0.040)	−0.133 (0.040)	−0.116 (0.040)	−0.106 (0.040)	−0.130 (0.041)	−0.122 (0.040)
	*0.015*	*0.019*	*0.024*	*0.029*	*0.023*	*0.028*	*0.008*	*0.021*	*0.034*	*0.010*	*0.015*
Gender (% male)	−0.018 (0.825)	−0.002 (0.831)	−0.083 (0.842)	−0.016 (0.821)	0.014 (0.821)	0.005 (0.825)	0.031 (0.863)	−0.015 (0.792)	−0.016 (0.783)	−0.014 (0.8088)	0.021 (1.298)
	0.731	0.976	0.125	0.761	0.786	0.930	0.570	0.774	0.743	0.792	0.802
BMI (kg/m^2^)	−0.072 (0.107)										
	0.169										
Waist (cm)		−0.110 (0.042)									0.072 (0.077)
		*0.039*									0.432
Percentage of body fat (%)			−0.117 (0.078)								
			*0.029*								
Systolic blood pressure (mmHg)				−0.077 (0.026)							
				0.148							
Diastolic blood pressure (mmHg)					−0.162 (0.036)						−0.129 (0.040)
					*0.002*						*0.024*
Mean arterial pressure (mmHg)						−0.133 (0.034)					
						*0.012*					
HDL−C (mg/dl)							0.161 (0.032)				0.127 (0.036)
							*0.004*				*0.036*
Fasting glucose (mg/dl)								−0.135 (0.017)			−0.085 (0.018)
								*0.008*			0.112
HbA1c (%)									−0.165 (0.496)		
									*0.001*		
Triglyceride (mg/dl)										−0.101 (0.004)	
										0.052	

The β (SE) and the *P* value for each factor in the models are shown, and the *P* values shown in italics text are significant (*P* < 0.05)

Moreover, the effects of the drug treatment on these variables were also considered. The treatment for hypertension, DM or hyperlipidemia was respectively used as the independent variable with the adjustment of age and gender in linear regression analyses, and it was observed that RNase-L was associated with the treatment for hypertension treatment (β = −0.121, *P* = 0.020, Table 3, Model 1) or hyperlipidemia (β = −0.107, *P* = 0.038, Table 3, Model 2), but not with that for DM (β = −0.040, *P* = 0.442, Table 3, Model 3). We further adjusted the hypertension treatment for DBP and MAP models (in Table 2, Models 5 and 6). The DBP was still negatively related to serum RNase-L levels (β = −0.137, *P* = 0.014, Table 3, Model 4), but the relation with MAP was only of a borderline significance (β = −0.103, *P* = 0.067, Table 3, Model 5). In both these two regression models, the associations between RNase-L and hypertension treatment were not significant (β = −0.076, *P* = 0.162 and β = −0.087, *P* = 0.114, respectively in Table 3, Model 4 and 5). If we adjusted the treatment of hyperlipidemia for HDL-C (in Table 2, Model 7), both HDL-C and the treatment of hyperlipidemia were still independently related to RNase-L (HDL-C: β = 0.157, *P* = 0.004; hyperlipidemia treatment: β = −0.102, *P* = 0.046, Table 3, Model 6). Whereas when treatment of hyperlipidemia was adjusted for TG (in Table 2, Model 10), both TG and the treatment of hyperlipidemia were only of a borderline significance (TG: β = −0.089, *P* = 0.088; hyperlipidemia treatment: β = −0.096, *P* = 0.063, Table 3, Model 7) in relation to RNase-L levels. Taken together, the adjustment with these treatments did not significantly alter the results in Table 2.

**Table 3:** The relations among serum RNase-L, metabolic factors, and treatments in multivariate linear regression analyses

Variables	Model 1 β (SE) *P* value	Model 2	Model 3	Model 4	Model 5	Model 6	Model 7
Age (year)	−0.090 (0.041)	−0.099 (0.041)	−0.111 (0.041)	−0.097(0.041)	−0.092 (0.041)	−0.114 (0.041)	−0.111 (0.041)
	0.080	0.053	*0.032*	0.060	0.073	*0.026*	*0.031*
Gender (% male)	−0.023 (0.788)	−0.025 (0.789)	−0.036 (0.788)	0.016 (0.821)	0.006 (0.824)	0.043 (0.864)	−0.004 (0.809)
	0.655	0.621	0.477	0.761	0.903	0.439	0.933
Hypertension treatment (%)	−0.121 (0.976)			−0.076 (1.029)	−0.087 (1.035)		
	*0.020*			0.162	0.114		
Hyperlipidemia treatment (%)		−0.107 (1.221)				−0.102 (1.210)	−0.096 (1.227)
		*0.038*				*0.046*	0.063
DM treatment (%)			−0.040 (1.417)				
			0.442				
Diastolic blood pressure (mmHg)				−0.137 (0.038)			
				*0.014*			
Mean arterial pressure (mmHg)					−0.103 (0.036)		
					0.067		
HDL-C (mg/dl)						0.157 (0.032)	
						*0.004*	
Triglyceride (mg/dl)							−0.089 (0.004)
							0.088

The β (SE) and the *P* value for each factor in the models are shown, and the *P* values shown in italics text are significant (*P* < 0.05)

When the waist circumference, DBP, HDL-C and fasting plasma glucose were used as the independent variables after adjusting age and gender in multivariate linear regression analysis, only DBP and HDL-C were significantly associated with RNase-L serum levels (Table 2, Model 11). However, if the HbA1c was applied as the independent variable instead of the fasting plasma glucose in the model, there was a significant independent relation between HbA1c and the levels of serum RNase-L (β = −0.126, *P* = 0.018, data not shown). Interestingly, age had a negative relation with serum RNase-L level throughout these analyses (Table 2).

Consistently, when the five diagnostic components of MetS were respectively set as the independent variable in linear regression analyses, central obesity (β = −0.138, *P* = 0.006), IFG (β = −0.113, *P* = 0.029), the diagnosis of MetS (β = −0.102, *P* = 0.045), and the count of MetS components (β = −0.169, *P* < 0.001) were negatively related to serum RNase-L levels after adjusting age and gender (Table 4, Model 2, 5, 7 and 8, respectively). The components of elevated BP (β = −0.095, *P* = 0.070) and low HDL-C (β = −0.089, *P* = 0.077) were also of borderline significance with that of RNase-L in regression analysis (Table 4, Model 3 and 4, respectively). If these two significant components, central obesity and IFG, were further applied as the independent variables in multivariate linear regression analysis, only central obesity (β = −0.125, *P* = 0.014) was significantly associated with RNase-L serum levels (Table 4, Model 9).

**Table 4:** The relation between serum RNase-L and the diagnosis of MetS in multivariate linear regression analyses

Variables	Model 1 β (SE) *P* value	Model 2	Model 3	Model 4	Model 5	Model 6	Model 7	Model 8	Model 9
Age (year)	−0.119 (0.040)	−0.116 (0.040)	−0.100(0.041)	−0.125 (0.040)	−0.095 (0.041)	−0.126 (0.041)	−0.114 (0.040)	−0.103 (0.040)	−0.077 (0.041)
	*0.019*	*0.021*	0.053	*0.013*	0.065	*0.013*	*0.023*	*0.039*	0.060
Gender (% male)	−0.039 (0.785)	−0.062 (0.790)	−0.019 (0.801)	−0.039 (0.783)	−0.026 (0.787)	−0.017 (0.815)	−0.021 (0.795)	−0.017 (0.782)	−0.049 (0.795)
	0.442	0.221	0.710	0.437	0.603	0.744	0.686	0.740	0.334
Central obesity		−0.138 (0.768)							−0.125 (0.773)
		*0.006*							*0.014*
Elevated blood pressure			−0.095 (0.833)						
			0.070						
Low HDL-C				−0.089 (0.829)					
				0.077					
Impaired fasting glucose					−0.113 (0.897)				−0.095 (0.900)
					*0.029*				0.066
Hypertriglyceridemia						−0.079 (0.927)			
						0.132			
Diagnosis of MetS							−0.102 (0.877)		
							*0.045*		
MetS component count								−0.169 (0.277)	
								<*0.001*	

The β (SE) and the *P* value for each factor in the models are shown, and the *P* values shown in italics text are significant (*P* < 0.05)

In binary logistic regression models, we respectively estimated the odds ratios (OR) for the MetS and its five diagnostic components with every 5 μg/ml change in serum RNase-L concentration with the adjustment of age and gender. With every 5 μg/ml increase in serum RNase-L concentration, the risk of MetS (OR 0.83, 95% CI 0.71–0.98, *P* = 0.028), central obesity (OR 0.82, 95% CI 0.71–0.94, *P* = 0.005), or low HDL-C (OR, 0.86, 95% CI 0.74–1.00, *P* = 0.042) was reduced by 14–18% (Table 5). The risk of elevated BP, IFG and hypertriglyceridemia also tended to be lower in the subjects with a higher serum RNase-L levels, however, all with a borderline statistical significance (Table 5).

**Table 5:** Odds ratios (95% CI) for MetS according to every 5 μg/ml serum RNase-L increase

Variables	Odds ratio	*P* value	95% confidence interval (CI)
			Lower–upper
Diagnosis of MetS	0.83	*0.028*	0.71–0.98
Central obesity	0.82	*0.005*	0.71–0.94
Elevated blood pressure	0.87	0.066	0.75–1.01
Low HDL-C	0.86	*0.042*	0.74–1.00
Impaired fasting glucose	0.85	0.054	0.71–1.00
Hypertriglyceridemia	0.86	0.080	0.73–1.02

Age and gender were adjusted in all odds ratio estimations

Odds ratio were estimated upon every 5 μg/ml RNase-L increase

Italics text indicates the statistically significant difference (*P* < 0.05)

## Discussion

The worldwide epidemic of metabolic syndrome (MetS) has become a major health issue which particularly addresses on the prediction, diagnosis, and treatment of metabolic disorders, such as obesity, type 2 DM (T2DM), cardiovascular diseases [1, 2]. The definition of MetS is the presence of any 3 of 5 risk components, including elevated waist circumference, BP, fasting glucose, TG, and reduced HDL-C that are respectively related to abdominal obesity, pre-hypertension, pre-diabetic condition, and hyperlipidemia [24]. Obesity has been recognized as a “disease” over the past decades, the inseparable relations of which with the driving of many metabolic disorders and dysfunctions are also raised [30]. It is worth noting that only visceral adiposity, but not subcutaneous, showed a high correlation with cardiometabolic risk and MetS [31, 32]. Accumulation of visceral fat in obese individuals altered the expression profile of inflammation-related genes in peripheral blood cells, suggesting that is also related to MetS via chronic inflammation [33]. Moreover, it was also documented that MetS is a cluster of interrelated and heritable traits that contributed to the disclosure of genetic risk factors [34].

For many years, RNase-L has been a focus of investigation as a key factor of innate immunity, especially in interferon responses to viral infection [13]. However, increasing number of studies have uncovered the diverse biological functions of RNase-L [14]. Meanwhile, RNase-L was ubiquitously expressed not only in the immunological tissues, but also in the metabolic tissues, such as the adipose tissues and skeletal muscles [16, 21]. Although it may potentially participate in many pathological processes, its relation with human diseases is rarely investigated. The most explored human disease associated with RNase-L so far is prostate cancer. Several single-nucleotide polymorphisms (SNPs) of the human RNase-L gene were reported to associate with hereditary and sporadic prostate cancer [35]. However, the mechanisms underlying these associations are unclear at present. In this report, we showed that lower serum RNase-L levels were associated with metabolic syndrome and related metabolic disorders.

The roles of RNase-L in metabolic disorders were best investigated in rodents by Bisbal’s group [21, 22]. Using mouse embryo fibroblasts deficient of RNase-L, they reported that RNase-L controlled adipocyte differentiation via regulating the expression of CHOP-10, a negative regulator of adipogenesis [21]. Surprisingly, the RNase-L knockout mice had expanded adipose tissues probably caused by adipocyte hyperplasia. These animals had ectopic fat deposition in liver and kidney. We recently also reported that knockdown of RNase-L reduced 3T3-L1 adipocyte differentiation and lipid accumulation [20]. Up-regulation of Pref-1, a well-known adipogenesis inhibitor, could explain this adipogenic impairment. We showed that Pref-1 mRNA is a substrate and specifically degraded by RNase-L activity [20].

Indeed, the alterations in the cellular composition and properties of adipose tissue could be one of the major causes of metabolic dysregulation during obesity and aging [36, 37]. In fact, the human genetic polymorphisms of several adipogenic factors, such as adiponectin and peroxisome proliferator-activated receptor γ (PPARγ), are also associated with adipogenesis and insulin sensitivity which lead to the modulations of adipose tissue development, obesity, and diabetic conditions [38, 39]. The reduced serum RNase-L levels in humans might be similar to the case of adiponectin. Hypo-adiponectinemia is the result of insulin resistance in adipose tissue, which is capable of mediating the metabolic effects on peripheral metabolic tissues [40–42]. The agonists of PPARγ have been widely applied for the management of hyperglycemia in T2DM by its effects on insulin sensitivity and serum adiponectin increase, and adipocyte differentiation induction that often results in weight gain [43, 44]. The reduced RNase-L levels may work likewise to prevent further weight gain in subjects already with the MetS. However, these speculations require further investigation.

Moreover, the Bisbal’s group further investigated the role of RNase-L in skeletal muscles [22]. They found that over-expression of RNase-L in C2C12 mouse myoblast cells enhanced insulin signaling pathway in a palmitate-induced insulin resistance model [22]. Most interestingly, the RNase-L level was not changed but the upstream OAS was reduced in the myotubes from obese insulin resistant human subjects compared with that from obese insulin sensitive subjects. Therefore, the activation of RNase-L was found defective in the myotubes isolated from the obese insulin resistance subjects. Taken together, reduced RNase-L activity in rodents appeared to associate with increased adipose tissues with ectopic fat deposition and increased insulin resistance in skeletal muscles. These mechanistic explorations in rodents and humans seem to support our findings that reduced serum RNase-L level is associated with the MetS and related metabolic disorders.

The studies in rodents by Bisbal et al. discussed above may well explain the association of the MetS, visceral obesity, hypertriglyceridemia, and impaired fasting glucose with reduced serum RNase-L levels. Moreover, some mutations in the human gene RNase-L were reported to associate with familial elevated HDL-C [45]. Although the mechanism underlying this relation remains unclear, there might be a direct link between HDL-C and RNase-L. As for the association between elevated BP and reduced RNase-L remains elusive and warrant further investigation.

One consistent finding in our regression analyses was that RNase-L was negatively associated with age. Three decades ago, it was already reported that 2-5A synthetase and binding protein activities (presumably RNase-L) were significantly reduced in peritoneal macrophages from older guinea pigs than those from the young ones [46]. It was also documented that the amount and the activity of RNase-L in the liver were lower in old rats than that of the young adults [47]. These phenomena are implicated to confer the altered responses with aging to environmental changes, especially the susceptibility to viral infection [47]. With regard to the metabolic phenotypes, whether the decline of serum RNase-L with age may contribute to insulin resistance, sarcopenia, obesity and the MetS in elderly population remains to be answered. In contrast, it was also reported that experimental animals with RNase-L deficiency had longer life span than the wild-type animals [48]. But it is not clear whether these animals can actually live a similar long life in natural environment. Moreover, it was also documented that the RNase-L protein level of mouse muscle was negatively associated with proteasomal activity which is responsible for the turnover of proteins and decreased with age as well [49, 50]. As a result of that, the transgenic mouse with a weaker proteasomal chymotrypsin-like activity had higher RNase-L protein level, shorter life span, and the susceptibility to obesity [50]. Therefore, the mechanism and the implication of the reverse association between serum RNase-L levels and age warrant further exploration, it may be also involved in the control of age-related protein turnover and obesity.

To evaluate the variance of general serum protein expression between MetS and non-MetS subjects, we calculated the difference of serum total protein (TP, sum of albumin and globulin) levels between MetS and non-MetS subjects as the control, and found it was similar (7.3 ± 0.4 vs. 7.2 ± 0.4 g/dl, *P* = 0.357, Table 1). In Fig. 1, it was shown that the subjects with MetS components had lower serum RNase-L levels. It is noted that the difference of serum RNase-L levels in female subjects was no longer significant (Fig. 2). However, in the multivariate linear regression analyses, gender was not associated with serum RNase-L whether after adjusting age (Table 4, Model 1), or age and MetS (Table 4, Model 7). Thus, it might be explained by a small sample size of only 26 female MetS subjects recruited. In addition, our study is also limited by the cross-sectional design, a further longitudinal investigation is needed.

In type I IFN-stimulated innate immune axis, OAS, RNase-L, and retinoic acid-inducible gene 1 (RIG-1) are often mentioned together as antiviral proteins [13]. In recent years, the diverse extracellular roles of them have begun to be discussed. The levels and activities of serum OAS can be up-regulated by IFN stimulation, which was related to the antiviral effect on the treatment of hepatitis C patients [51, 52]. It was also demonstrated that extracellular OAS1 can trigger the antiviral activity by entering into the cellular cytoplasm even in the RNase-L-null cells [53]. Moreover, extracellular OAS2 was documented to be capable of down-regulating the T-cell receptor chain (CD3-ζ) via caspase-3 activation, thereby contributing to a decrease in T-cell responsiveness [54]. The plasma level of RIG-1, as an RNase-L downstream molecule, were found to be higher in patients with mild cognitive impairment, suggesting that extracellular RIG-1 level may be involved in the incipient neurodegenerative disorders, such as Alzheimer’s disease [55]. In this study, we reported the presence of RNase-L in human serum and analyzed its relation with MetS, but the functions of these antiviral proteins in extracellular compartment remain unclear. OAS, RNase-L, and RIG-1 are all cytosolic proteins that have no signal sequence, hence, the release process could not be the conventional trafficking pathways of endoplasmic reticulum and Golgi complex. The secretion of RIG-1 from macrophages was enhanced by the activation of caspase-1, a component of ‘inflammasome’ complex [56]. Moreover, RNase-L also plays a crucial role in activating the nucleotide-binding oligomerization domain-like receptor 3 (NLRP3) inflammasome [57]. It is possible that RNase-L as well could be delivered into a subset of secretory lysosomes via forming a complex with inflammasome [58]. In addition, extracellular protein may also be released by cell lysis which somehow is in part triggered by MetS [59]. But contradictorily, serum RNase-L level was lower in the MetS subjects, thus we speculate the RNase-L release is unlikely be the result of cell lysis.

Collectively, this is to our knowledge one of the earliest investigation of human serum RNase-L and an interesting and significant inverse relation between its level and the MetS, various related metabolic factors and age was observed. These links were at least in part supported by previous cell and animal mechanistic experiments. Since RNase-L is a key factor in innate immunity, our observation may provide a hint about the complex interactions among inflammation, metabolism and aging.

## Conclusions

Our results showed that serum RNase-L levels were inversely associated with MetS, unfavorable metabolic profiles and age, suggesting that serum RNase-L could be a potential biomarker for the diagnosis of MetS.

## Abbreviations

MetS: metabolic syndrome
DM: diabetes mellitus
RNase-L: ribonuclease L
OAS: oligoadenylate synthetase
RIG-1: retinoic acid-inducible gene 1
2-5A: ppp5′A[2′p5′A]n
IFN: interferon
MyoD: myogenic differentiation-1
HuR: Hu antigen R
TTP: tristetraprolin
Pref-1: pre-adipocyte factor-1
CEBP: CCAAT/enhancer-binding protein
CHOP10: CCAAT/enhancer-binding homologous protein 10
ELISA: enzyme-linked immunosorbent assay
IDF: International Diabetes Federation
AHA/NHLBI: American Heart Association/National Heart, Lung, and Blood Institute
TBST: Tris-buffered saline with Tween-20
f.c.: final concentration
TMB: 3, 3′, 5, 5′-tetramethylbenzidine
OD: optical density
CV: coefficients of variability
CI: confidence interval
BMI: body mass index
HbA1c: hemoglobin A1c
HDL-C: high-density lipoprotein cholesterol
MAP: mean arterial pressure
OR: odds ratio
SNPs: single-nucleotide polymorphisms
PPARγ: Peroxisome proliferator-activated receptor γ
